# Robots Show Us How to Teach Them: Feedback from Robots Shapes Tutoring Behavior during Action Learning

**DOI:** 10.1371/journal.pone.0091349

**Published:** 2014-03-19

**Authors:** Anna-Lisa Vollmer, Manuel Mühlig, Jochen J. Steil, Karola Pitsch, Jannik Fritsch, Katharina J. Rohlfing, Britta Wrede

**Affiliations:** 1 Centre for Robotics and Neural Systems, School of Computing and Mathematics, University of Plymouth, Portland Square, Plymouth, United Kingdom; 2 Honda Research Institute Europe, Offenbach/Main, Germany; 3 Faculty of Technology, Research Institute for Cognition and Robotics, Bielefeld University, Bielefeld, Germany; Arizona State University, United States of America

## Abstract

Robot learning by imitation requires the detection of a tutor's action demonstration and its relevant parts. Current approaches implicitly assume a unidirectional transfer of knowledge from tutor to learner. The presented work challenges this predominant assumption based on an extensive user study with an autonomously interacting robot. We show that by providing feedback, a robot learner influences the human tutor's movement demonstrations in the process of action learning. We argue that the robot's feedback strongly shapes how tutors signal what is relevant to an action and thus advocate a paradigm shift in robot action learning research toward truly interactive systems learning in and benefiting from interaction.

## Introduction

If robots are to become ubiquitous helpers in our society, they need to be able to learn about actions relevant for new tasks and environments that they have to cope with. Innumerable possible situations a robot could encounter render the research field of imitation learning particularly important because it aims at replacing manual programming by learning from a tutor's demonstration [1]. This requires the detection of a tutor's action demonstration and its relevant parts [2]. It is a persistent research question how tutors convey the meaning of actions and which factors control their demonstrations, even when some regularities can be identified: In child-directed interaction, for example, tutors modify their body movements to direct the learners' attention [3], [4], [5]. Recent research suggests that in both robot- and child-directed interaction, tutors modify their linguistic and nonverbal behavior to act appropriately for their analysis of the learner's understanding and the resulting communicative situation [6]. The meaning of an action can be highly person specific and depends on the history that the tutor has with that action. Consider, for example, to drive in a nail with a hammer. The goal is to drive the nail home without bending and without hitting one's own finger. Depending on the tutor's own experience (and expertise level) s/he will focus on the aspect s/he deems most important in order to not overload the pupil's cognitive capabilities. We therefore use the term “tutor's knowledge” – instead of the term “action type” which suggests an objective and universal meaning of actions – to emphasize the subjective nature of action meaning.

**Figure 1:**
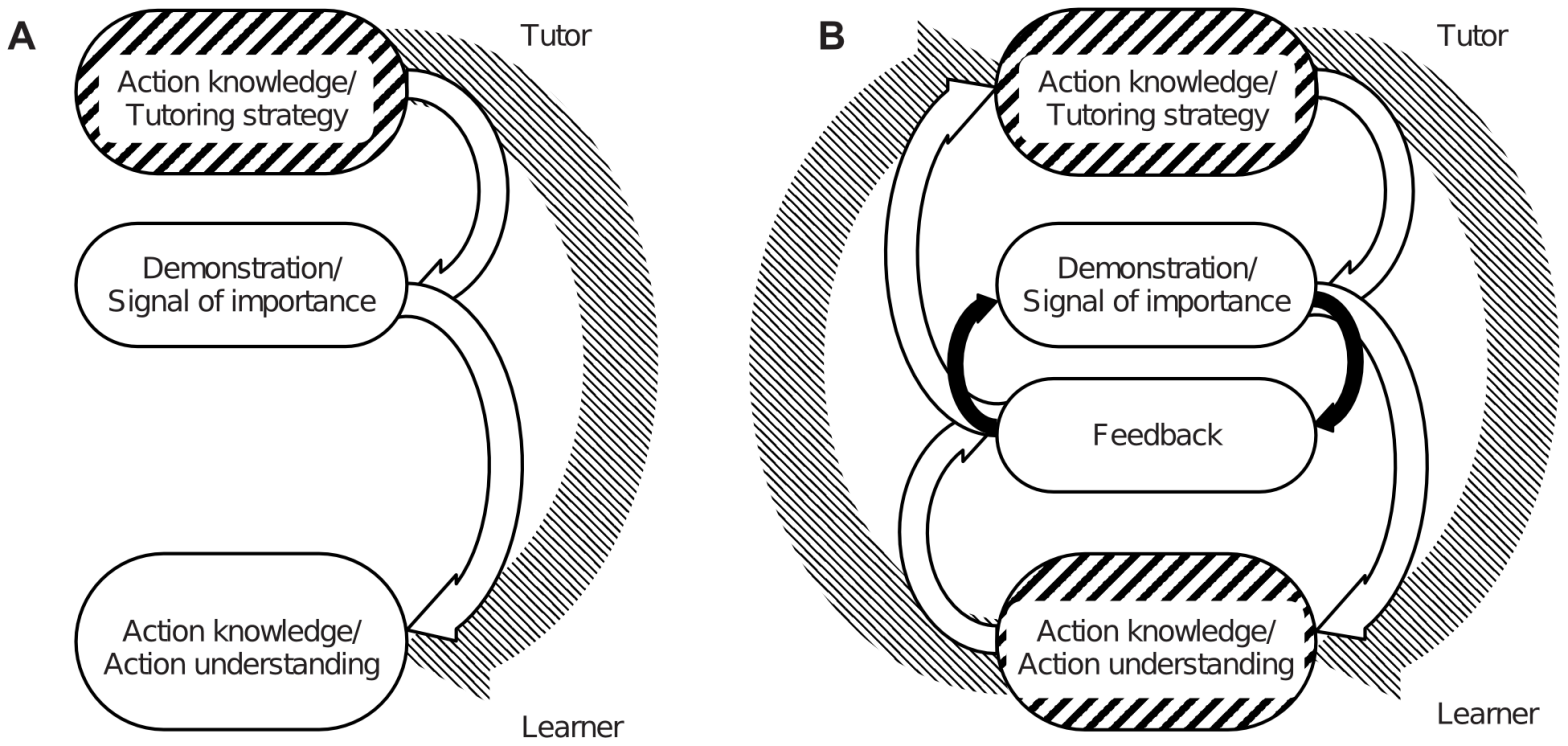
Action learning concept graphics. (A) Unidirectional concept of current imitation learning approaches: The tutor demonstrates the action (white oval) according to his/her knowledge (upper hatched oval). The learner passively observes the action demonstration and learns the action. (B) Interactionist concept of learning: The tutor demonstrates the action (upper white oval) corresponding to his/her knowledge (upper hatched oval) emphasizing what is relevant to the action accordingly. The learner's level of understanding or knowledge of the action (lower hatched oval) is communicated by his/her feedback (lower white oval). This feedback directly influences the tutor's action demonstration. The tutor monitors the learner's feedback, builds hypotheses about the learner's understanding, and reacts by changing his/her demonstration accordingly as will be shown in this contribution.

Traditional approaches of action learning in robots – which implicitly assume a unidirectional transfer of action knowledge from tutor to learner [2] (Figure 1 A) – specify what is relevant to an action to the robot beforehand (either manually or by defining expected, usually artificial, tutor behaviors that signal movement relevance). They are therefore limited to one or a few specific tasks. In real-world scenarios with untrained users, however, this is not realistic. On a trajectory level, probabilistic approaches can model which parts of simple manipulatory tasks are relevant by gaining information from the variance over multiple demonstrations of the same movement [7], [8]. These approaches, however, have not been evaluated with untrained users.

There exists a limited amount of related work not only in the field of imitation learning which is considering a bi-directional interaction between tutor and learner. Thomaz et al. introduce learner feedback with what they term “transparency mechanism” into machine learning systems. These approaches include action learning by a reinforcement learning agent in a web-based setup [9]. The agent uses a fixed set of actions on a fixed set of objects. Before an action, it gives feedback by gazing at all objects involved in those actions the system deems relevant next. Thus, the system's uncertainty about the following action is proportional to the amount of time gazing and a corresponding delay before the next action is carried out. Only during this phase the tutor could provide guidance about the next relevant action to the learner by drawing a yellow square outline onto an object with the right mouse button. Additionally the tutor could provide a positive or negative numerical reward to the learner at any time during the interaction.

Another symbolic approach for learning object affordances by exploration involves a robot learner communicating that the object to be learned is too far or too close by tilting its neck to the upper limit and back [10]. The only social signal the human tutor could provide was to choose an object from a set, horizontally center it in the robot's workspace when the robot had its arms in an idle position and decide how often the robot should manipulate it.

A number of current dialog systems also incorporate feedback from the robot learner [11], [12], [13]. For instance, for compound symbol learning, the authors of [11] employed non-verbal robot feedback in form of a fixed sequence of behaviors and a set of animations to communicate a certain object and the confidence in an answer, respectively. In this setup, the tutor presented the symbols and provided information or queried the system by saying three possible predefined sentences. In a study presented in [12] the content of questions posed by a robot was varied to investigate its influence on responses from the human partner for object recognition. Another study implemented verbal and non-verbal feedback in a robot to investigate its influence on itinerary requests [13].

These works try to approach the issue from a different angle than the work at hand. They use symbolic systems in restricted interactions, where it is straightforward to incorporate social cues and system feedback and investigate their influence on the learning mechanism. In these approaches for the most part, the important cues are predefined. Learning methods using symbolic encoding rely on a large amount of prior knowledge, so preprogrammed interaction protocols are employed. In contrast, we are aiming at examining in complex natural interaction how robot feedback influences the tutor's behavior which provides social cues about the meaning of actions.

Recently, an approach to learning continuous movement skills has been proposed which integrates social cues from the tutor (i.e. prosody, head orientation and gaze direction), though it has not been tested with inexperienced users [14]. We are not aware of related work on imitation of actions on a trajectory-level which incorporates robot feedback into the system.

The presented work challenges the predominant assumption of a unidirectional knowledge transfer based on an extensive user study with an autonomously interacting humanoid robot. We subscribe to a perspective present in research in social human-human interaction emphasizing the process of alignment between mental states, actions' goals [15], and communication [16]. Correspondingly, action learning via interaction has the aim to align the learner's mental states or action goals to those of the tutor in a co-construction, which is not possible through active perception only (active perception refers to strategies involving for instance an autonomous re-positioning of the robot's sensors to increase information gain and improve perception [17]). Subscribing to the interactive view in Human-Robot Interaction (HRI), it is not the user alone who determines what is being demonstrated (Figure 1A) (as it is currently implicitly assumed in robot imitation learning) but the demonstration has to emerge with the feedback of the learner (Figure 1 B).

Feedback behavior is essential since, as we have previously shown in parent-child interactions [3], tutors are highly sensitive to the learner's feedback, which is an important cue to infer the learner's current state of understanding. Parents for instance modify their manual movements with regard to their child's focus of attention. Also robots – similarly to children – can benefit from the input tailored specifically to their perceptional and cognitive capabilities [6]. In current HRI, the interactive view on social cognition and communication has not been tested, because most robots are barely capable of a real interaction. An appropriate setting requires endowing a humanoid robot with autonomous feedback behavior, which is a technically demanding task which we had to solve to conduct the current study (see Methods section). This is opposed to commonly applied Wizard-of-Oz techniques, in which a human operator remotely controls the robot, making them much simpler but unsuitably implying generating human feedback instead of robotic feedback for the robotic system [18].

Adopting the interactive view, we argue that it is both the tutor's knowledge about the action to be transferred to the learner (H1) and also the feedback behavior of the robot (H2 and H3) that determines the tutor's demonstrations. Concretely, our hypotheses are:

1. H1. Action demonstrations differ depending on the tutor's semantic knowledge about an action.

To control for the action knowledge which is conveyed to the robot, we designed two different kinds of tasks: goal- and manner-crucial [19]. This aspect of action understanding, which the tutor wants to transfer to the learner, refers to the importance of the goal state of an action versus the importance of the manner in which an action is carried out.

1. H2. The robot's feedback influences future action demonstrations of the tutor.

As often emphasized, it is necessary for the tutor to monitor the level of understanding of the learner [20]. The robot learner's way of replicating an action is therefore an important *turn-based feedback* giving the tutor cues with respect to the robot's action understanding. A human learner's understanding of an action evolves from a rudimentary and holistic representation to a rich and structured one [21]. Thus, the tutors who are all unaware of learning methods in artificial systems will try to deduce the system's action representation from its feedback behavior, and react accordingly. In particular, we hypothesize that the tutor will repeat the demonstration of an action in a modified way, if the robot executes the action incorrectly. However, the tutor will be satisfied with the robot's performance and will not repeat the demonstration, if the robot executes the action correctly.

1. H3. The robot's feedback directly influences the tutor's current demonstration.

The robot's gaze during the tutor's action demonstration serves as an important *online feedback*. Similarly to (H2), it provides the tutor with cues with respect to action understanding.

## Materials and Methods

### Ethics Statement

The Technical Faculty of Bielefeld University does not have an ethics committee dealing with human-robot interaction research. Nevertheless, this research was conducted in accordance with the ethical principles for human subject research expressed in the Declaration of Helsinki. For the described analyses only anonymous data were used. Partially informed consent was obtained in writing from all subjects participating in the study. Subjects were told the robot would try to replicate their movements, even though the robot behavior was predetermined according to the feedback condition (see next section). A debriefing session, in which all research methods and aims were fully disclosed to the subjects, followed the study.

### Subjects

In the current study, 59 adult subjects (28 m, 31 f) were instructed to teach a full-size humanoid robot equipped with a fully autonomous feedback behavior (see the technical setup below) how to perform specific actions with eight different objects. One subject was excluded from all analyses because she neglected the task instructions. The subjects were right-handed to avoid side differences in action presentation, they were German native speakers to avoid language-based differences in action presentation, and they did not have any experience with robots (The majority of subjects had some experience working with computers, *M* = 3.42, *SD* = 1.06 on a scale of 1 [no experience] to 5 [very much experience], but subjects indicated that they had minimal to no experience interacting with robots. *M* = 1.24, *SD* = 0.5 on the same scale.). The study was gender-balanced and subjects were equally distributed across four age groups (20–30 years, 30–40 years, 40–50 years and above 50 years). Additionally, equal gender balanced numbers of subjects from each age group were randomly assigned to three robot gazing behavior conditions.

### Setup and Conditions

**Figure 2:**
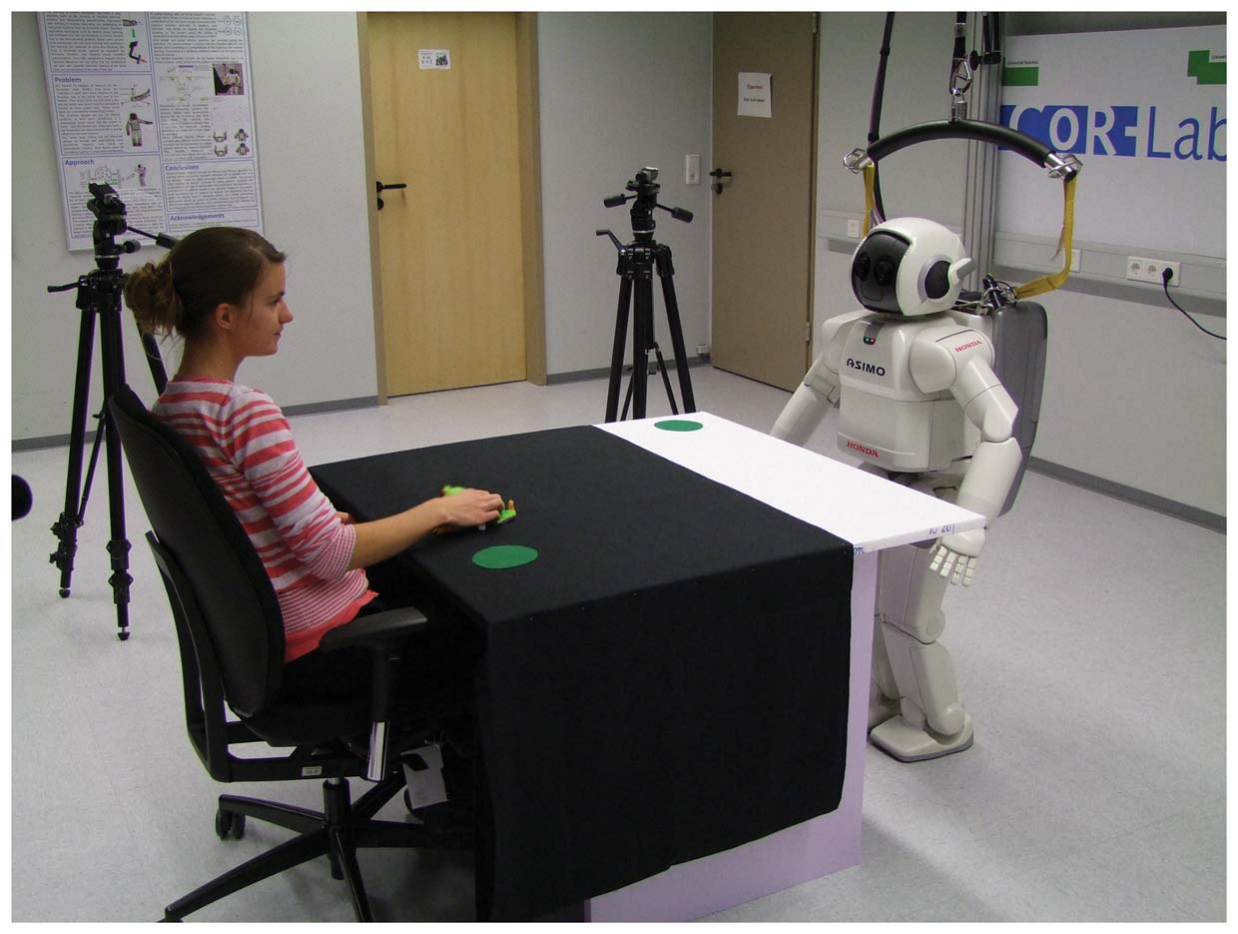
Experimental setting. Human tutor is sitting across from the robot learner at a table. Green marks on the table indicate the starting points for both the tutor's and the robot's demonstrations. **Note that the individual in this Photograph (** **Figure 2** **) has given written informed consent (as outlined in PLOS consent form) to publication of her photograph.**

**Figure 3:**
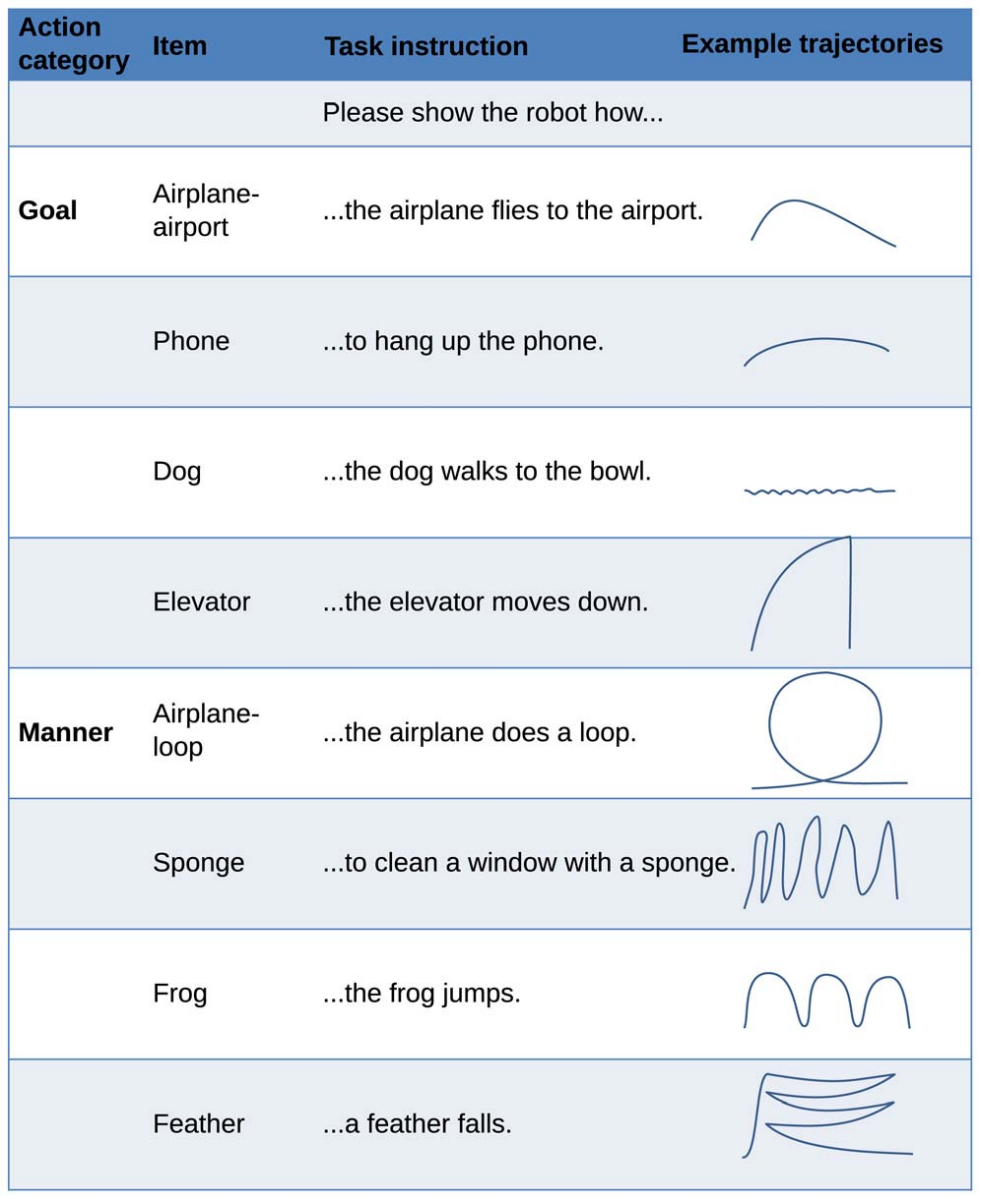
Items, task instructions, and example trajectories.

Each subject sat in front of the standing humanoid robot with a table of 1 m width in between and had to present eight different object manipulation actions to the robot (Figure 2, Figure 3, Movie S1). These actions were simple everyday actions all subjects were familiar with and were chosen to result in comparable executions of the actions across subjects. They fell into two categories: manner-crucial, and goal-crucial actions. In manner-crucial actions, the manner and path are the most important features of the action. As for example for the task to show how to clean a window with a sponge, the movements are important and not where the sponge is set down. For goal-crucial actions in contrast, the goal position of the object is important, rather than how it got there. For example, when a phone is hung up, it is important that the handset is on the hook at the end, but it does not matter if it reaches this position in a curved or straight movement.

To test the effect of the robot's replication behavior (turn-based feedback) (H2), the robot replicated the observed action according to the two main ways of replicating a movement in human children and apes: imitation and emulation [22]. These two ways have been identified to manifest action understanding [19]. Imitation involves copying of the path and manner of the action and reproducing the goal state. Emulation, in contrast, involves the achievement of the goal state without copying the path and manner of the movement. Thus, after the tutor's turn of demonstrating an action, the robot reacted either with a correct or an incorrect replication behavior depending on the category of action. In detail, for manner-crucial actions imitation should be a correct replication behavior and emulation an incorrect replication behavior. In contrast for goal-crucial actions emulation should be a correct replication behavior, whereas imitation should be an incorrect replication behavior because imitation involves the copying of incidental behavior which is unnecessary for the demonstrated action. From the eight actions to be reproduced for each subject, four actions (of which two were manner-crucial and two were goal-crucial) were imitated by the robot, i.e. it reproduced the trajectory of the object as exactly as possible (Movie S1); four (of which analogously two were manner-crucial and two were goal-crucial) were emulated, i.e. the robot reproduced the end state only with a straight, goal-directed movement (Movie S1) (for the technical realization, please refer to the section on the technical setup below). After the robot's replication of the action, the subject could choose to demonstrate the action again and the robot reproduced the action once more, forming an interaction loop, which repeated until the subject decided to stop. The robot replicated a certain action always in the same way (either imitation or emulation) and for that action did not change its replication behavior.

To test the effect of online feedback (H3), each subject was presented with one of three robot eye gaze behaviors (see below and Movie S1): (a) social gaze to simulate action understanding and attention and (b) random and (c) static gaze as control conditions. For online feedback, we chose a between-subjects design to prevent subjects' experience in one condition from affecting the subjects' behavior in subsequent conditions. Task order and actions belonging to a reproduction condition were randomized within the above constraints. During interactions we tracked object movements and employed these trajectories to compute objective measurements about movement properties (see section on computational measures below). After the eight tasks had been completed, the subjects filled out a questionnaire and were interviewed.

#### Online Feedback Conditions and Interaction Details

The experiment involved three robot gaze behavior conditions: Social gaze, random gaze, and static gaze. The robot's gaze was initially pointed at a fixed scene position (i.e., a point between the face of the tutor and the table).

The *social* robot gaze behavior was designed to reflect the learner's behavior observed in adult-child tutoring interactions [23]. The robot either exhibited attentive gaze following the object movements or anticipating expected end positions of the transported object. The object was initially set down at the participant's start position by the experimenter. At this point, the robot shifted its gaze toward the object. During the participant's action demonstration, the robot gave continuous online feedback by following the moving object with its gaze depending on the turn-based feedback condition:

- Imitation: The robot followed the object with its gaze, until the subject had finished the action demonstration (Movie S1).
- Emulation: The robot followed the object with its gaze for two seconds and then switched its gazing direction toward a predefined end position, anticipating where the object should be set down (Movie S1).

At the specific point in time, right after the participant's task demonstration was complete, the robot initiated the replication by gazing at the tutor's face and then to the object, while reaching out its right arm in the direction of the object. After that, the robot followed the object, until the experimenter placed it into the robot's hand. Then the robot began the action replication. The social gaze condition additionally included a behavior after the robot replicated the action. While setting down the object on the table after the action replication was complete, the robot gazed at the object and after that at the tutor encouraging the tutor to react to the shown replication.

For the *random* gazing condition, the robot's gaze had five directions between which it alternated beginning when the object was set down at the start position (Movie S1). The duration of the gaze intervals and probability of occurrence of a specific direction were designed to follow random distributions modelled after 12 to 24 months old children's gaze directions during action demonstrations in parent-infant interactions. The intervals and gaze directions were investigated and corresponding statistics calculated on an existing corpus of video recorded adult-child and adult-adult interactions [4]. In a semi-experimental setting, parents were asked to present a set of manipulative tasks both to their infant and to another adult. The fix points of the children's gaze behavior were annotated and divided into four classes, of which only three were considered and their likelihood was calculated.

1. Gaze to object: 88.41% to cover all relevant positions of the tutoring situation and task, this figure was divided into three equally distributed classes for the robot:
  - Object: 29.47%.
  - Start position: 29.47%.
  - End position: 29.47%.
2. Gaze to tutor's face: 10.87%.
3. Gaze to tutor's stationary hand: 0.72%.
4. Gaze elsewhere: The fourth class of all gaze anywhere other than to the object, the parent or the stationary hand was not taken into account because the random gaze condition aimed at controlling the timing of gaze to relevant positions, but was not designed to include gaze to positions entirely irrelevant to the task, which - independent of the timing of gaze - trigger attention getters at any given moment.

For the duration of gaze intervals to each of the three gazing directions, log-normal distributions were fit to the histograms of the data obtained from the corpus to serve as probability distributions for the modelled random gaze behavior (see Figure S1). The log-normal distributions have the following parameters:

1. Gaze to object (equal for all sub-classes): μ = −0.246, σ = 0.926.
2. Gaze to parent's face: μ = −0.586, σ = 0.772.
3. Gaze to tutor's stationary hand: μ = −0.455, σ = 0.711.

After the participant's action demonstration, the robot gazed to the fixed scene position between the table and the tutor's face and initiated the replication by lifting its arm to reach for the object. Concerning the end of the robot's replication, in this gazing condition, the robot gazed to the fixed scene position as well when releasing the object.

In the *static* gazing condition, the robot maintained the fixed scene gazing direction at all times (Movie S1). This direction was chosen between the face of the tutor and the height of the starting point of the task, such that the tutor had the impression the robot had witnessed the demonstration. After the action demonstration, the robot's gaze remained unchanged as it reached for the object to initiate the replication. The robot also gazed to the fixed scene position when releasing the object at the end of the robot's replication in this gazing condition.

For the time during which the robot replicated the movement, no constraints were imposed on the robot's head movements. This allowed the robot to utilize more of its degrees of freedom, which resulted in smoother and more natural movements.

#### Technical Setup

**Figure 4:**
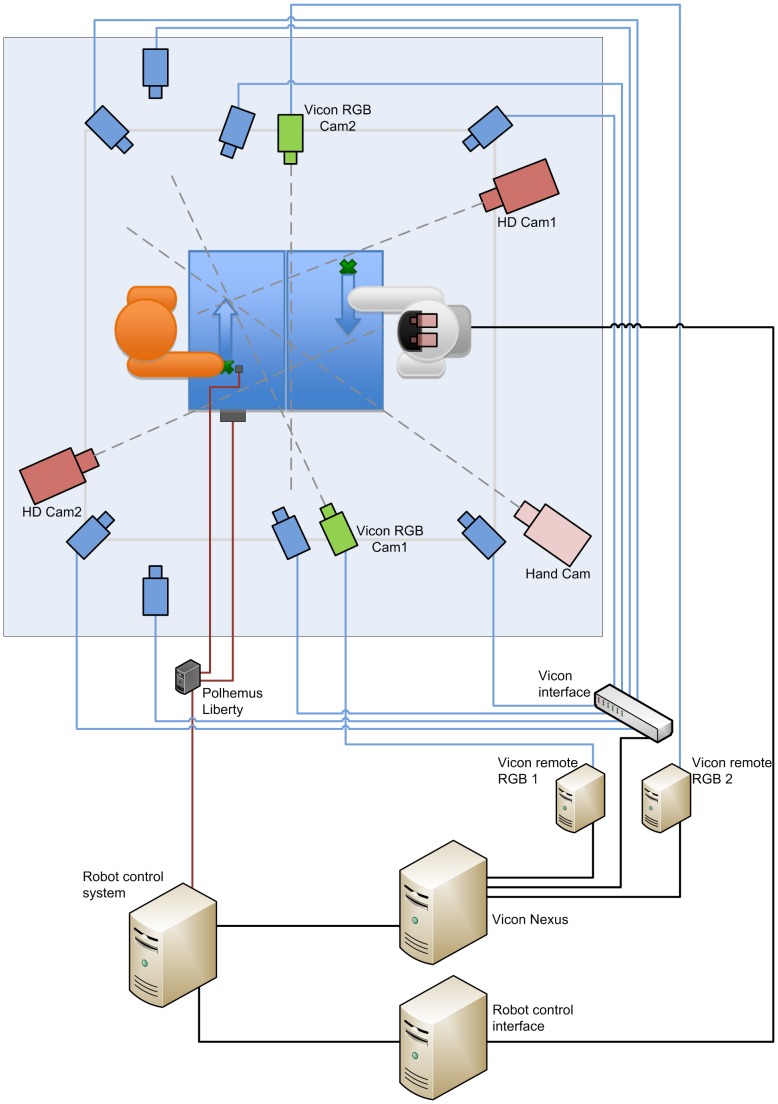
Technical setup. For sensing the subject's movements, a Vicon system with 8 IR cameras (blue) was used. Additionally the object's position was tracked using a Polhemus Liberty magnetic-field-based tracking system (dark grey). This information was fed into the “robot control system” for generating appropriate robot behavior. For evaluating the study, additional data was recorded by 2 RGB cameras (green) directly synchronized with Vicon, 2 RGB cameras in the robot's head, 2 high-definition cameras (red) and an additional simple hand camera (light red) for showing interesting scenes during the interview after the study.

Figure 4 shows the technical setup of the conducted study. As described earlier, subjects had to demonstrate certain actions and the robot either imitated (precise reproduction of the observation) or emulated (only end-point reproduction with a straight movement) the observed action. Note that instead of remote controlling the robot, all of its behavior was generated autonomously based on the subject's actions. This approach makes the setup more realistic in terms of actual HRI compared to typically employed Wizard-of-Oz remote control. For this, we used a state machine mechanism based on earlier work [7]. This state machine was set up such that certain actions of the subject trigger certain robot behaviors and thus advance the interactive sequence. We recorded the required information for doing so using a Vicon motion capture system (for recording the subject's hand and head poses) and a Polhemus Liberty magnetic-field-based tracking system (for recording the object position). This information was fed directly into the robot control system, which generated control commands for the robot.

Specifically, the start of a demonstration was identified by the robot based on the object moving away from the start position (distance to start position and velocity above certain thresholds). The end of the demonstration was defined by the object being located on the table, not moving and the subject's hands moving away from it (height of object position and object velocity below certain thresholds, and distance from the object to the subject's right and left hand above a certain threshold). The trajectory of the object was recorded between these key points and constituted the detected action.

In the imitation feedback condition the demonstrated trajectory was rotated (180° around the vertical axis) and clipped to fit the robot's working range so that the velocity of the demonstration remained the same. In the emulation condition only the end point of the recorded trajectory was used and the robot moved the object straight to this point with a predefined velocity. As the focus of this user study is on imitation and emulation as forms of feedback, no learning was involved (unlike in [7]). As stated earlier the robot replicated each action always in one way (either imitation or emulation) and did not change its replication behavior for the same action.

For evaluating the study, we recorded extensive data:

- The complete robot state including joint space configuration and state machine state.
- The subject's head and hand positions and orientations.
- The object position and orientation.
- Video material (2 Vicon RGB cameras, 2 HD cameras, 2 robot on-board cameras, a separate hand camera).

### Computational Measures

**Figure 5:**
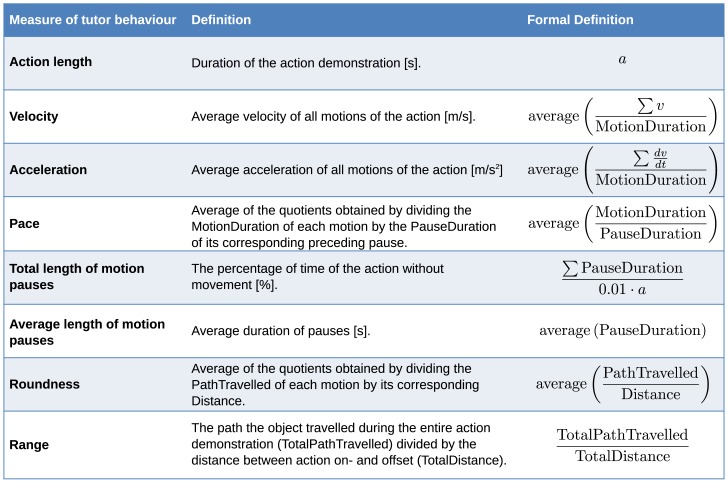
Definitions of measures of tutor behavior. A visual depiction for the measure roundness can be found in Figure S4 of the Supporting Information.

To be able to assess the behavior modifications in the tutors' demonstrations, in MATLAB, we counted the number of times each action was demonstrated to the robot. Additionally, we calculated quantitative measures for movement properties on the trajectory data for which we utilized the tracked object positions obtained via the Polhemus Liberty system. The measures have proven to reveal important modifications in tutoring behavior in earlier work [4], [24]. We segmented the data stream into motions and pauses based on the object velocity and the direction of movement. For a sequence segmented as a motion, the path the object travelled (PathTravelled) and the distance between start and end point (Distance) were calculated. Additionally, the duration of each motion (MotionDuration) and pause (PauseDuration) was measured in seconds. The measures for movement properties of the tutor behavior are described in the table in Figure 5. All measures were computed for all objects and averaged over the four goal-crucial actions on the one hand and the four manner-crucial actions on the other hand.

## Results

The following results from statistical analyses show that indeed both factors, the subjects' action knowledge and the robot's feedback, determined how the subjects demonstrated the actions.

### Action Knowledge (H1)

**Figure 6:**
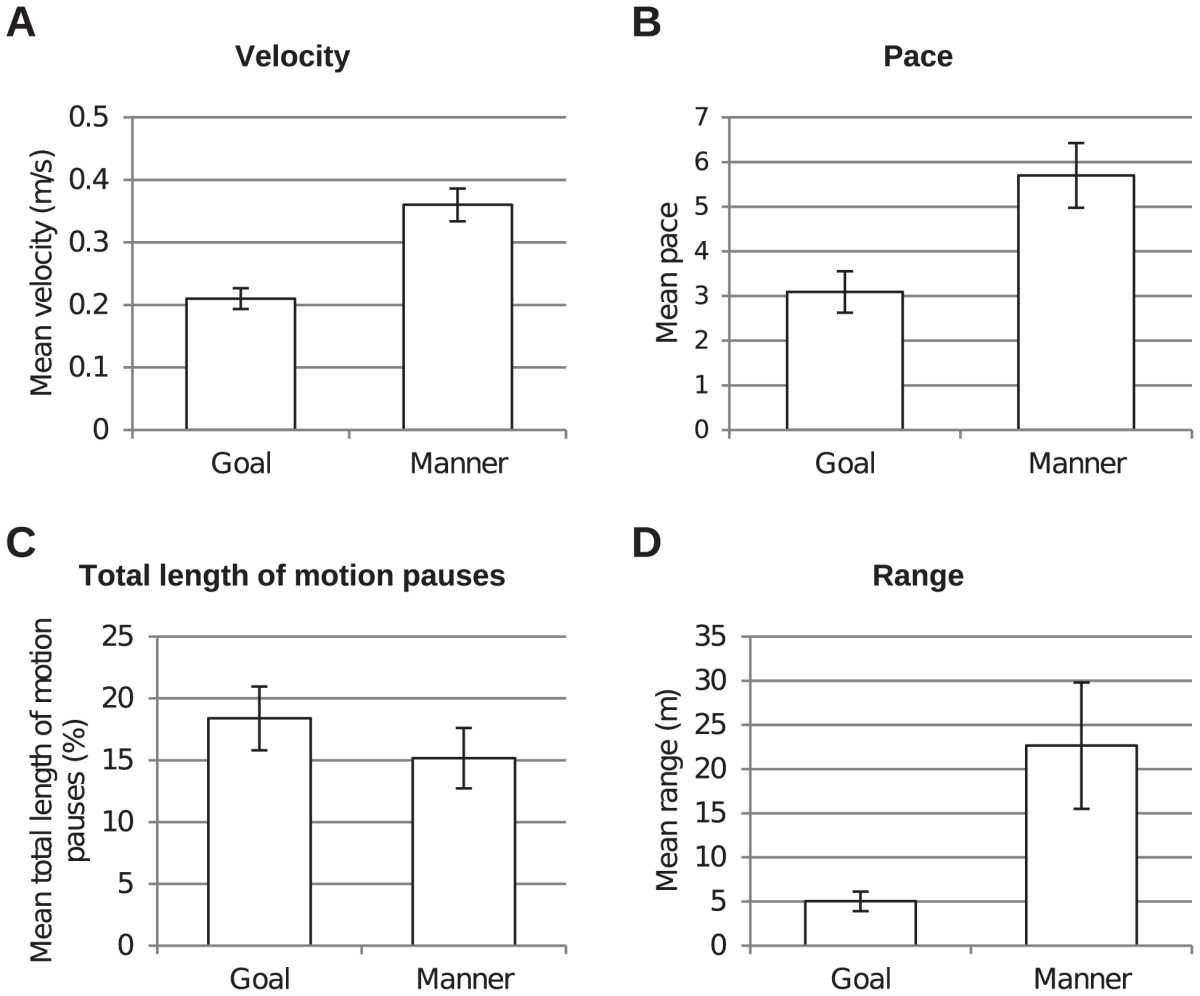
Mean values for movement measures as a function of action knowledge condition. Paired-sample *t* tests (df = 53) revealed significant differences between conditions for the presented movement measures, (A), velocity (*t* = −16.11, *p*<0.001), (B), pace (*t* = −6.81, *p*<0.001), (C), total length of motion pauses relative to action length (*t* = 2.47, *p* = 0.017), and (D), range (*t* = −5.1, *p*<0.001). Additionally, the measures action length, acceleration, and roundness revealed significance. Error bars represent standard errors.

The subjects' action knowledge significantly influences the way in which they demonstrate actions (Figure 6). Manner-crucial actions were demonstrated significantly longer, faster, with rounder movements, and significantly more range.

The subjects' action knowledge was assessed using a paired-samples t-test on the movement property measures calculated on the first demonstration of each object and averaged over the four goal-crucial actions on the one hand and the four manner-crucial actions on the other hand. The action knowledge associated with the object movement (goal- or manner-crucial) was considered a within-subject factor in the repeated measures design. We included only the tutors' first demonstrations of each object in this analysis because they were not influenced by the robot's turn-based feedback, yet.

Several significant differences have been found (Table 1). The action length differed significantly across the two groups: goal (*M* = 6.58 s, *SD* = 2.24) and manner (*M* = 9.81 s, *SD* = 3.9), *t*(55) = −8.41, *p*<0.001. Thus, the subjects demonstrated manner-crucial actions longer than goal-crucial actions.

**Table 1:** Statistical results of action knowledge influence on tutor behavior.

Action knowledge						
Measure of tutor behavior	Action category: Goal		Action category: Manner			
	*M*	*SD*	*M*	*SD*	*t*	*p*
Action length	6.58	2.24	9.81	3.9	−8.41	0.000
Velocity	0.21	0.06	0.36	0.1	−16.11	0.000
Acceleration	1.1	0.38	2.01	0.7	−13.17	0.000
Pace	3.09	1.66	5.7	2.62	−6.81	0.000
Total length of motion pauses	18.39	9.52	15.17	9.03	2.47	0.017
Average length of motion pauses	0.47	0.25	0.44	0.25	1.01	0.317
Roundness	1.43	0.43	3.46	1.39	−10.28	0.000
Range	5.02	3.96	22.66	26.64	−5.1	0.000

In addition the same was revealed concerning the speed of the demonstrations. Manner-crucial actions (*M* = 0.36 m/s, *SD* = 0.1) were carried out faster than goal-crucial actions (*M* = 0.21 m/s, *SD* = 0.06), velocity (Figure 6A): *t*(55) = −16.11, *p*<0.001, with higher acceleration (manner-crucial: *M* = 2.01 m/s^2^, *SD* = 0.7, goal-crucial: *M* = 1.1 m/s^2^, *SD* = 0.38), *t*(55) = −13.17, *p*<0.001, and with higher pace (manner-crucial: *M* = 5.7, *SD* = 2.62, goal-crucial: *M* = 3.09, *SD* = 1.66), *t*(55) = −6.81, *p*<0.001 (Figure 6B).

For the total length of motion pauses, the manner-crucial actions (*M* = 15.17%, *SD* = 9.03) were demonstrated with less pauses than the goal-crucial actions (*M* = 18.39%, *SD* = 9.52), *t*(55) = 2.47, *p*<0.05 (Figure 6C). The manner-crucial actions (*M* = 3.46, *SD* = 1.39) were also carried out with less roundness than the goal-crucial actions (*M* = 1.43, *SD* = 0.43), *t*(55) = −10.28, *p*<0.001.

For the average length of motion pauses, we did not find any significant differences between manner-crucial actions (*M* = 0.44 s, *SD* = 0.25) and goal-crucial actions (*M* = 0.47 s, *SD* = 0.25), *t*(55) = 1.01, *p* = 0.317.

For range the results show that subjects demonstrated manner-crucial actions (*M* = 22.66, *SD* = 26.64) with a higher range than goal-crucial actions (*M* = 5.02, *SD* = 3.96), *t*(55) = −5.1, *p*<0.001 (Figure 6D).

### Turn-based Feedback (H2)

**Figure 7:**
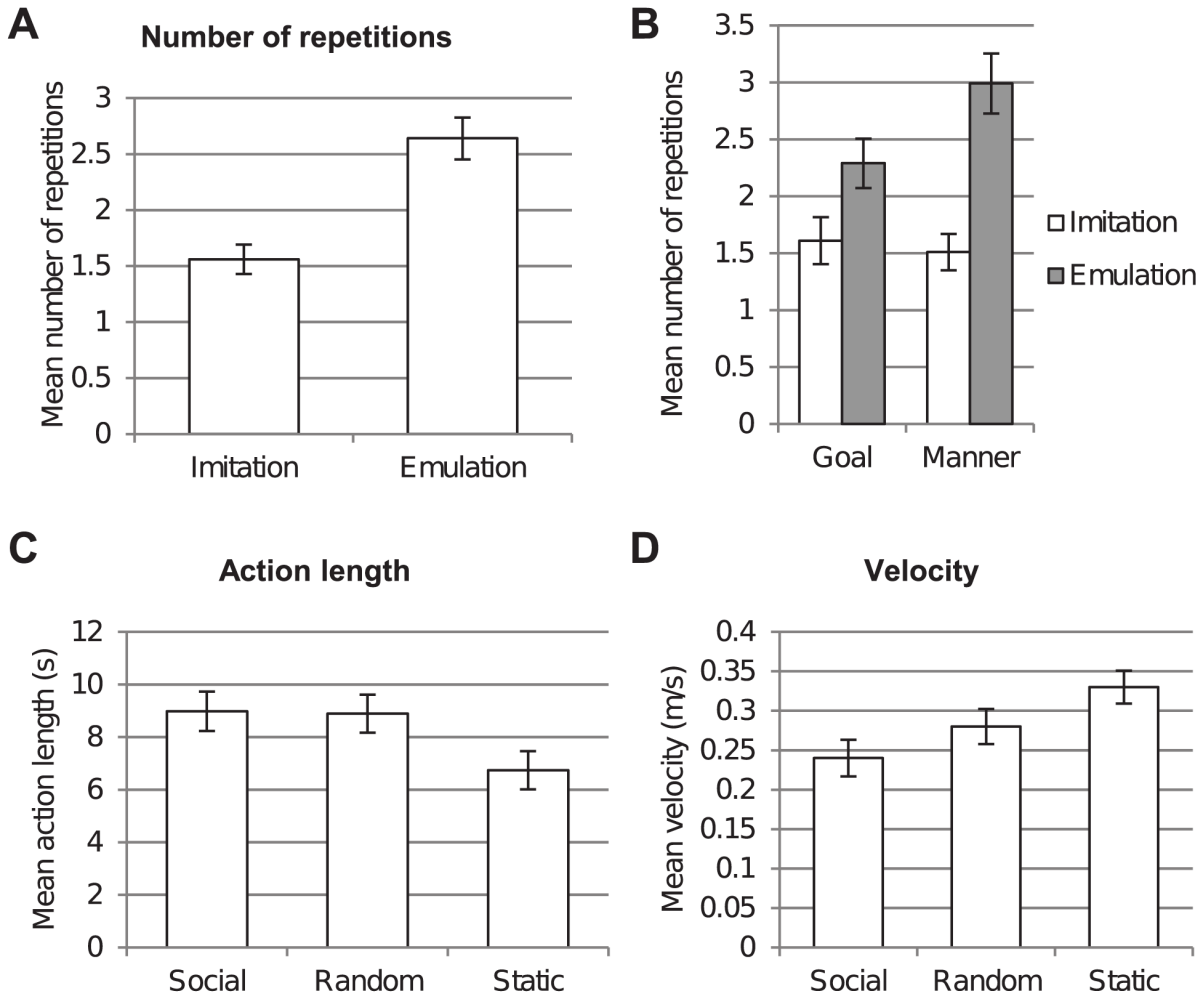
Influence of robot feedback on tutors' action demonstrations. (A and B), Mean values for number of repetitions as a function of turn-based feedback condition and action knowledge. A two-way repeated measures ANOVA (df = 40) revealed a significant interaction effect (Λ = 0.71, *F* = 16.23, *p*<0.001) between conditions, (A) a main effect for turn-based feedback (Λ = 0.22, *F* = 140.93, *p*<0.001), and a main effect for action knowledge (Λ = 0.71, *F* = 16.45, *p*<0.001). (B) A Scheffé post hoc comparison indicated that the turn-based feedback effect was greater in the manner-crucial action knowledge condition than in the goal-crucial condition. Error bars represent standard errors. (C and D) Mean values for movement measures as a function of online feedback. A one-way between subjects ANOVA (df = 53) revealed significant differences between gaze conditions for the presented movement measures, (C) action length (*F* = 4.18, *p* = 0.021) and (D) velocity (*F* = 7.302, *p* = 0.002). Scheffé tests used to make post hoc comparisons between conditions revealed that subjects in the social gaze condition demonstrated significantly slower and longer than in the static gaze condition (velocity: *p* = 0.002, action length: *p* = 0.049). The comparison between the other groups did not reveal any significant results. Additionally, significance was found for the measure acceleration. Error bars represent standard errors.

The robot's turn-based feedback determines how often a subject demonstrates an action (Figure 7, A and B). Emulated actions were shown significantly more often than imitated ones. Manner-crucial actions, which were emulated by the robot, were demonstrated a higher number of times. Also emulated goal-crucial actions were demonstrated more often than imitated goal-crucial actions, however not significantly.

A two-way repeated measures ANOVA was conducted to compare the effect of robot's turn-based feedback behavior and the tutor's action knowledge on the number of the tutor's demonstrations in 2 (imitation, emulation) ×2 (goal, manner) conditions. Results revealed a significant interaction effect (Λ = 0.71, *F* = 16.23, *p*<0.001) and a significant main effect of robot's feedback behavior on the number of times the tutor repeated the demonstration, Wilks' Lambda, Λ = 0.22, *F*(1,40) = 140.93, *p*<0.001 (Table 2). According to the main effect, subjects repeated the demonstration more often, when the robot emulated (*M* = 2.64, *SD* = 0.75) than when it imitated (*M* = 1.56, *SD* = 0.57) the action (Figure 7A). Further, the test revealed another main effect for action knowledge (Λ = 0.71, *F* = 16.45, *p*<0.001) which indicated more repetitions for manner-crucial actions (*M* = 2.25, *SD* = 0.66) than goal-crucial actions (*M* = 1.95, *SD* = 0.66). A Scheffé post hoc comparison indicated that the feedback effect was greater in the manner-crucial action knowledge condition than in the goal-crucial condition and revealed that the highest number of demonstrations was carried out, when a manner-crucial action was presented, which the robot emulated (*M* = 2.99, *SD* = 0.83) (Figure 7B). All comparisons between conditions revealed significance (*p*<0.001), except the comparison between imitated goal-crucial and imitated manner-crucial actions (*p* = 0.96). Additionally, to protect against violating the assumption of normality, we applied a logarithmic transformation to the variables and obtained results of the same significance.

**Table 2:** Statistical results of turn-based feedback influence on number of action demonstrations (repetitions).

Turn-based Feedback							
Measure of tutor behavior	Learner behavior: Imitation		Learner behavior: Emulation				
	*M*	*SD*	*M*	*SD*	*Λ*	*F*	*p*
Number of repetitions	1.56	0.57	2.64	0.75	0.22	140.93	0.000

The first demonstration of an action, especially of the first action, is a demonstration which is not influenced by the turn-based feedback and thus can be considered a base-line condition of the turn-based feedback. In [25], we investigated the first demonstrations of the first action and compared it to the tutors' subsequent second demonstrations of the same action after the robot's first replication. The main findings indicate that subjects particularly emphasized important aspects of the action in a second demonstration, when the robot showed a correct replication to some extent. When the robot replicated the action incorrectly, subjects simplified their second demonstrations. With respect to the results presented here, this shows that tutors adapt their action presentations according to the robot's turn-based feedback.

### Online Feedback (H3)

The robot's online feedback in terms of gaze behavior had an impact on the tutor's on-going action demonstration (Figure 7, C and D). When the robot was in the social gaze condition, the subjects demonstrated the actions significantly slower compared to the static gaze condition.

The effect of the online feedback behavior on the movement properties of the demonstration the tutor carried out, was considered using a one-way between subjects ANOVA in the social gaze, random gaze, and static gaze conditions. Here, also, only the first demonstrations of the actions were considered.

There was a significant effect of robot's online feedback on the velocity and acceleration of the presentation, velocity: *F*(2,53) = 7.302, *p*<0.01 and acceleration: *F*(2,53) = 8.824, *p*<0.001 (Figure 7D) (Table 3). A Scheffé test was used to make post hoc comparisons between conditions. It revealed that subjects in the social gaze condition demonstrated significantly slower (velocity: *M* = 0.24 m/s, *SD* = 0.08, acceleration: *M* = 1.27 m/s^2^, *SD* = 0.53) than in the static gaze condition (velocity: *M* = 0.32 m/s, *SD* = 0.07, acceleration: *M* = 1.87 m/s^2^, *SD* = 0.53), velocity: *p*<0.01 and acceleration: *p*<0.01. For velocity, the comparison between the other groups did not reveal any significant results (random gaze: *M* = 0.28 m/s, *SD* = 0.13; compared to social gaze *p* = 0.22, compared to static gaze *p* = .125). For acceleration the test uncovered that subjects in the random gazing condition (*M* = 1.51 m/s^2^, *SD* = 0.39) also demonstrated with a lower acceleration than subjects with static robot gaze (*M* = 1.87 m/s^2^, *SD* = 0.53), *p*<0.05. We did not find significant differences between the social and the random gaze conditions, *p* = 0.268.

**Table 3:** Statistical results of online feedback influence on movement properties.

Online feedback								
Measure of tutor behavior	Learner's gaze: Social		Learner's gaze: Random		Learner's gaze: Static			
	*M*	*SD*	*M*	*SD*	*M*	*SD*	*F*	*p*
Action length	8.98	2.98	8.89	3.45	6.74	2.23	4.18	0.021
Velocity	0.24	0.08	0.28	0.13	0.32	0.07	7.302	0.002
Acceleration	1.27	0.53	1.51	0.39	1.87	0.53	8.824	0.000
Pace	4.71	1.77	4.37	1.54	4.24	1.37	0.45	0.64
Total length of motion pauses	17.33	8.74	18.16	8.8	16.42	5.24	0.232	0.794
Average length of motion pauses	0.43	0.15	0.53	0.28	0.4	0.15	2.255	0.115
Roundness	2.37	0.89	2.79	0.96	2.41	0.69	1.436	0.247
Range	14.56	13.26	14.17	11.48	12.01	9.48	0.26	0.772

Likewise, there was a significant effect of robot's online feedback on the action length of the presentation, *F*(2, 53) = 4.18, *p*<0.05 (Figure 7C) (Table 3). Again a Scheffé test was used to make post hoc comparisons between conditions. It revealed that subjects in the social gaze condition (*M* = 8.98 s, *SD* = 2.98) demonstrated significantly longer than in the static gaze condition (*M* = 6.74 s, *SD* = 2.23), *p*<0.05. Additionally, a statistical trend for subjects to demonstrate longer in the random gaze condition (*M* = 8.89 s, *SD* = 3.45) than in the static gaze condition was found, action length: *p* = 0.056, but no difference was found between the social and random gaze conditions, *p* = 0.995.

The other measures (pace, total and average length of motion pauses, roundness, and range) did not reveal any significant differences between conditions (please refer to Table 3 for details).

The questionnaire and interview results are presented and discussed in Text S1, Figure S2, and Figure S3 of the Supporting Information.

## Discussion

Summarizing our results, the presented study yields insights into how inexperienced tutors signal what is relevant about an action that they teach a learning humanoid robot. It revealed that the user's action demonstration strongly depends on the feedback that the robot gives. It is not the action knowledge of the tutor alone that shapes the tutor's action demonstration. Instead, it is also the feedback of the learner – in the form of action replication and eye gaze, indicating what has been understood – that influences repetition of action demonstration and modification of the tutor's movements.

In our study, we considered social, random, and static robot gaze behavior for online feedback. Our findings revealed that in the social gaze condition tutors demonstrated the actions slower than in the static gaze condition. This finding is in accordance with qualitative studies which have shown that tutors do not adapt their demonstrations to a robot with static gaze, but sequence their actions and adjust their movements on a micro-level to the robot's social gaze [26]. Slower demonstrations could be beneficial for the system's learning mechanism, as research on adult-child interaction suggests tutoring behavior toward infants to be slower than in adult-directed interaction [4], [24]. However, the results did not reveal any significant differences between the social and the random gaze behavior conditions. Whereas the random gaze behavior does not signal understanding, it seems to 'open a channel of communication' to the tutor, who uses it and adapts his demonstration accordingly. Measures concerned with the structure and form of the action (i.e., pace, total and average length of motion pauses, roundness, and range) seem similar in all three conditions.

We operationalized action knowledge as goal- vs. manner-crucial actions and tutors' action demonstrations differed according to these categories.

Concerning the robot's turn-based feedback, one important observation was that tutors repeated the imitated goal-crucial actions less often than the emulated goal-crucial actions suggesting that they considered it more correct when the robot imitated goal-crucial actions than when it emulated them. On the one hand, this fact could be explained by the tutor's tendency to omit unnecessary and incidental movements during the action demonstrations, which the robot would have reproduced in the imitation condition. On the other hand, this could indicate that subjects also pay close attention to the fine details of goal-crucial actions, which they interpreted as also involving a certain manner, which was reproduced by the robot in the imitation, but not in the emulation condition. That is, tutors generally tended to treat the imitation behavior of the robot as more correct than its emulation behavior, regardless of the action. This is in line with findings from Gergely & Csibra [27] who found that human infants as opposed to apes prefer imitation behavior [28]. They argue that imitation is a necessary capability when learning actions with goals that are opaque to the observer. Our results extend this insight by the observation that (a) human tutors also apply their teaching strategies to non-human entities such as robots and (b) there is an interactional loop, in which the tutor receives important additional information from the learner about how to design and specify subsequent demonstrations and explanations based on the received feedback.

## Conclusion

Robots can benefit from this information eliciting mechanism by actively modulating their feedback with the goal of reducing ambiguities in the tutor's demonstration. Due to the high amount of uncertainties in such demonstrations, and in order to keep the number of demonstrations reasonably low, it will be necessary to develop truly interactive learning systems that make use of social cues instead of only relying on statistical learning. Thus, we advocate the paradigm to consider an interactional loop for robot learning. According to our results, successful robot strategies for discriminating between goal- and manner-crucial actions and thereby finding what is relevant to a shown action entail (a) sensitivity to the signals of the tutor, such as movement speed, range or roundness of movements, and (b) active and explicit probing of hypotheses e.g. by deliberately emulating actions which leads to more distinctive tutor behavior for goal- vs. manner-crucial actions.

## Supporting Information

Figure S1 **Log-normal distributions for the three gaze directions.**
(TIF)

Figure S2 **Participants' tutoring strategies.** Percentages of participants reporting identified strategies in each robot gaze behavior condition in structured interview.
(EPS)

Figure S3 **Participants' perception of turn-based feedback.** Numbers of participants who reported respective categories positively and negatively in structured interview.
(EPS)

Figure S4 **Visual depiction for the measure roundness.** Example trajectories for the actions “how a frog jumps” and “how a feather falls” with high and low roundness values respectively.
(EPS)

Movie S1 **Experimental conditions.** The movie individually presents each condition of the two-by-two design formed by action knowledge (goal-, manner-crucial actions) and replication conditions (imitation, emulation) as well as the three online feedback conditions. Video clips show the original setup of the study and an actor demonstrating the actions to the robot. **Note that the individuals in this manuscript (Movie S1) have given written informed consent (as outlined in PLOS consent form) to publication of this video.**
(MP4)

Text S1 **Questionnaire and interview results.**
(DOCX)
