# Supplementary figures and images for: Robots Show Us How to Teach Them: Feedback from Robots Shapes Tutoring Behavior during Action Learning
Source: PLoS One. 2014 Mar 19;9(3):e91349. doi: 10.1371/journal.pone.0091349 (PMC3960110; doi:10.1371/journal.pone.0091349)

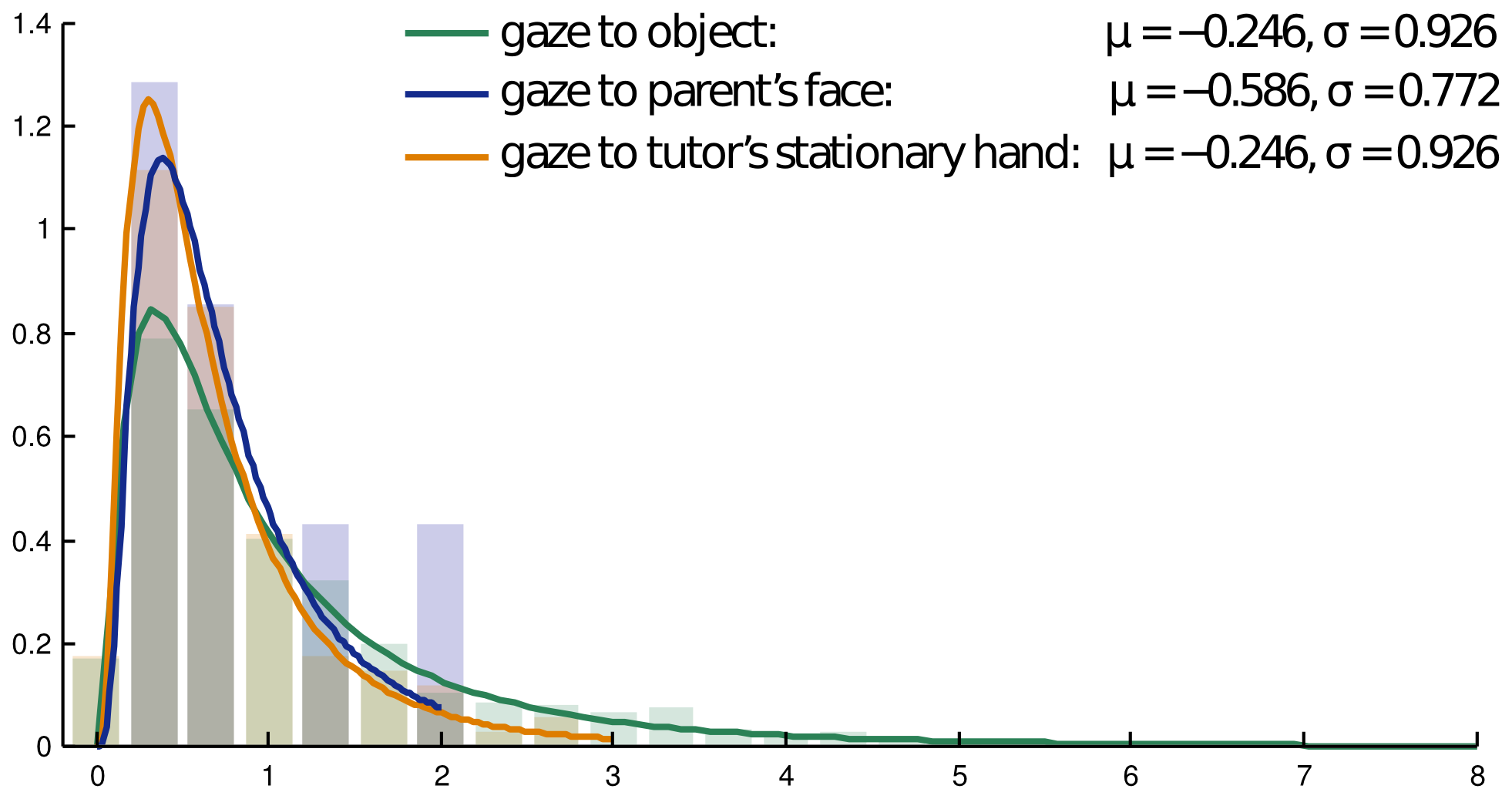

Supplement: Figure S1 — Log-normal distributions for the three gaze directions. (TIF) [file pone.0091349.s001.tif]
