# Supplementary material for: Robots Show Us How to Teach Them: Feedback from Robots Shapes Tutoring Behavior during Action Learning
Source: PLoS One. 2014 Mar 19;9(3):e91349. doi: 10.1371/journal.pone.0091349 (PMC3960110; doi:10.1371/journal.pone.0091349)
Supplement: Text S1 — Questionnaire and interview results. (DOCX) [file pone.0091349.s006.docx]

Text S1

Questionnaire and Interview Results

After the eight tasks had been completed, the subjects filled out a questionnaire and were interviewed (in German). The questionnaire and structured interview provided qualitative insights into subjects’ perception of the robot's feedback and resulting strategies to tutor the robot.

Online Feedback

The first analysis investigated how subjects perceived the robot’s online feedback. It utilized the answers to the question “Wohin hat der Roboter geguckt? (Where did the robot look?)”. Subjects’ answers could include multiple gaze targets. In a first step, we considered the direction each subject mentioned first and in a second step, we considered all directions mentioned. Subjects in the robot *social gaze* condition (N = 19) first mentioned only four different answers:

• The object (N = 11),

• the subject’s hand (N = 4),

• changing directions (N = 2)

• and the subject (N = 1).

One subject did not answer the question. Subjects in the condition in which the robot *gazed randomly* (N = 20) first mentioned seven different answers:

• The object (N = 7),

• changing directions (N = 4),

• the subject’s hand (N = 3),

• the subject (N = 3),

• the Vicon markers on the subject’s hand (N =1),

• the cable of the Polhemus marker (N = 1),

• and start and end position (N = 1).

Subjects where the robot had a *static gaze* direction (N = 19) first mentioned only three different answers:

• They did not pay attention to where the robot looked (N = 8),

• the subject (N = 6),

• and the table (N = 4).

Again one subject in this condition did not answer the question.

When we considered multiple answers for each subject, we found that in the *social gaze* condition with an average of 1.37 answers per subject, the answers ordered by the number of occurrences were

• the object (68.42%),

• the subject (36.84%),

• the subject’s hand (21.05%)

• and changing directions (10.53%).

In the *random gaze* condition with an average of 1.75 answers per subject, subjects answered

• The object (50%),

• the start or end positions (35%),

• the subject’s hand (25%),

• changing directions (20%),

• the subject (20%),

• the cable of the Polhemus marker (10%),

• and the Vicon markers on the subject’s hand (5%).

In the *static gaze* condition with an average of 1.31 answers per subject, subjects gave the answers

• they did not pay attention to where the robot looked (42.11%),

• the subject (42.11%),

• the table (26.32),

• straight (10.53),

• the object (5.26%),

• and the subject’s hand (5.26%).

Concerning the subjects’ strategy, the answers to the structured interview question “Worauf haben Sie bei der Interaktion geachtet? Hatten Sie eine Strategie? (What did you pay attention to during the interaction? Did you have a strategy?)” were investigated. In the *social gaze* condition, on average subjects (N =19) answered with 2.11 strategies. In total seven different strategies were mentioned first:

• Check robot gaze is attentive (N = 6),

• slower demonstrations (N = 5),

• check Vicon marker is in robot field of view (N = 2),

• simpler demonstrations (N = 2),

• check the robot's replication (N = 2)

• not to make movements with wrist (N = 1),

• show variations of the action (N = 1).

In the *random gaze* condition, one subject did not give a response to this question. On average subjects’ (N = 20) answers consisted of 2.15 strategies. As first mentioned strategy, we identified six different strategies.

• Slower demonstrations (N = 7),

• check the robot gaze is attentive (N = 3),

• not to use two hands (N = 3),

• more articulate demonstrations (N = 1),

• and move like a robot (N = 1).

In the *static gaze* condition, two subjects did not give an answer to the question. On average subjects (N = 19) answered with 1.58 strategies. As a first answer they indicated ten different strategies.

• Simpler demonstrations (N = 3)

• slower demonstrations (N = 3),

• more articulate demonstrations (N = 2),

• check Vicon marker is in robot field of view (N = 2),

• show variations of the action (N = 2)

• not to use two hands (N = 1),

• not to make movements with wrist (N = 1),

• higher demonstrations (N = 1),

• demonstrate like demonstrating to a child (N = 1),

• and smaller demonstrations (N = 1).

Considering all strategies given in subjects’ answers, an overview is provided with Figure S2 of the Supporting Information. In the *social gaze* condition, subjects answered

• slower demonstrations (47.37%)

• make sure the robot gaze is attentive (42.11%)

• simpler demonstrations (26.32%),

• Vicon marker in robot field of view (21.05%),

• and show variations of the action(15.79%).

In the *random gaze* condition, subjects stated

• slower demonstrations (60%)

• make sure the robot gaze is attentive (40%),

• Vicon marker in robot field of view (20%),

• do not use two hands (20%),

• and more articulate demonstrations (20%).

Subjects in the *static gaze* condition named

• slower demonstrations (36.84%),

• do not use two hands (21.05%),

• simpler demonstrations (21.05%),

• and more articulate demonstrations (15.79%).

Turn-based Feedback

A second analysis examined how subjects perceived the robot’s turn-based feedback. It utilized subjects’ answers to the question “Was von dem, was Sie ihm vorgemacht haben, hat der Roboter nachgemacht? (What of your demonstrations did the robot reproduce?)”. The answers were divided into positive and negative feedback about the experimental items and were grouped into one of the four categories of the 2 (goal, manner) x 2 (imitation, emulation) design. The results of this analysis are presented in Figure S3 of the Supporting Information. Subjects commented positively on 24 and negatively on only one imitated manner-crucial actions. They did not comment positively on emulated manner-crucial actions, but commented on eight such actions negatively. Concerning goal-crucial actions, subjects commented on two of the imitated actions and on four of the emulated actions positively. They commented on one negatively in each case.

Discussion

Our findings reflected that the robot’s social gaze was primarily perceived as attentive, the random gaze led subjects to think of reasons for the robot's inattentiveness and in the static gaze condition, subjects did not pay attention to the robot’s gaze.

Findings for the robot’s replications for manner-crucial actions are clear: Subjects perceived imitation to be the correct and emulation to be the incorrect replication behaviour. This is in line with the results for turn-based feedback (H2) that subjects demonstrated manner-crucial actions which were emulated by the robot a higher number of times.

According to subjects’ answers concerning their strategies, slower demonstrations is a comprehensive tutoring strategy in all robot gaze conditions, especially in the random gaze condition, suggesting that the robot gaze was thought to be the main reason for failed replication of the actions. In the social gaze condition, subjects additionally had the strategy to make demonstrations simpler assuming action executions to be too difficult. Other than in the social and random gaze conditions, in the static gaze condition the robot gaze was not involved in subjects’ many different strategies as cues about reasons for failed replications were missing. These findings, on the other hand, support the results obtained for online feedback (H3): In the social and random gaze condition, subjects demonstrated the actions significantly slower compared to the static gaze condition. First, slower demonstrations were indeed a strategy and second, the gaze of the robot was only considered in the social and random gaze condition, leading subjects to adapt their action demonstrations and perform the action slower (as opposed to the static gaze condition).
